# Safety of Single High-Dose Liposomal Amphotericin B for Induction Treatment of Cryptococcal Meningitis and Histoplasmosis in People With HIV: A Systematic Review and Meta-analysis

**DOI:** 10.1093/ofid/ofad472

**Published:** 2023-09-20

**Authors:** HeeEun Kang, John P Uy, Caroline C Ho, Heather B Blunt, Natalie B Riblet, Alessandro C Pasqualotto, Richard A Murphy

**Affiliations:** Infectious Disease and International Health, Department of Medicine, Dartmouth Hitchcock Medical Center, Lebanon, New Hampshire, USA; Geisel School of Medicine at Dartmouth, Hanover, New Hampshire, USA; Infectious Disease and International Health, Department of Medicine, Dartmouth Hitchcock Medical Center, Lebanon, New Hampshire, USA; Geisel School of Medicine at Dartmouth, Hanover, New Hampshire, USA; Infectious Disease and International Health, Department of Medicine, Dartmouth Hitchcock Medical Center, Lebanon, New Hampshire, USA; Geisel School of Medicine at Dartmouth, Hanover, New Hampshire, USA; Geisel School of Medicine at Dartmouth, Hanover, New Hampshire, USA; Geisel School of Medicine at Dartmouth, Hanover, New Hampshire, USA; Infectious Diseases, Universidade Federal de Ciências da Saúde de Porto Alegre, Porto Alegre, Brazil; Infectious Disease and International Health, Department of Medicine, Dartmouth Hitchcock Medical Center, Lebanon, New Hampshire, USA; Geisel School of Medicine at Dartmouth, Hanover, New Hampshire, USA; White River Junction VA Medical Center, Hartford, Vermont, USA

**Keywords:** HIV infection, antifungal, cryptococcal meningitis, histoplasmosis, opportunistic infections

## Abstract

**Background:**

Evidence for efficacy of single, high-dose liposomal amphotericin B (LAmB) in HIV-associated cryptococcal meningitis and histoplasmosis is growing. No systematic review has examined the safety of this regimen across multiple studies.

**Methods:**

We systematically searched Medline, Scopus, and the Cochrane Library from inception to April 2023 for studies reporting grade 3 and 4 adverse events (AEs) with single high-dose LAmB vs traditional amphotericin regimens for HIV-associated fungal infections.

**Results:**

Three trials (n = 946) were included. Compared with traditional regimens, single high-dose LAmB was associated with equivalent risk of grade 3 and 4 AEs (risk ratio [RR], 0.75; 95% CI, 0.53–1.06) and lower overall risk of grade 4 AEs (RR, 0.68; 95% CI, 0.55–0.86), grade 4 renal (RR, 0.43; 95% CI, 0.20–0.94) and grade 4 hematological AEs (RR, 0.46; 95% CI, 0.32–0.65).

**Conclusions:**

Single, high-dose LAmB is associated with a lower risk of life-threatening AEs compared with other World Health Organization–endorsed amphotericin B–based regimens in invasive HIV–related fungal infection.

Evidence for the efficacy of single high-dose liposomal amphotericin B (LAmB; AmBisome) in HIV-associated fungal infections is growing. In 2022, the World Health Organization (WHO) adopted single high-dose LAmB as the cornerstone of first-line induction treatment for HIV-associated cryptococcal meningitis [1] after the AMBITION trial showed noninferiority of this strategy [2]. Evidence is also emerging to support the efficacy of single high-dose LAmB as induction therapy for disseminated histoplasmosis in people with HIV [3].

LAmB is a lipid-based polyene formulation of amphotericin that received US Food and Drug Administration approval in 1997 [4]. LAmB has a favorable side effect profile compared with the older formulation of amphotericin (amphotericin B deoxycholate [DAmB]) [5] and is preferentially used in high-income countries. In resource-limited settings, access to LAmB is limited by high cost [6]. Yet the use of single high-dose LAmB induction treatment for cryptococcal meningitis carries potentially significant cost-saving benefits [7].

The AMBITION trial noted fewer grade 3 or 4 adverse events (AEs) in participants receiving single high-dose LamB compared with those receiving amphotericin B deoxycholate (DAmB) [2]. LAmB, despite being safer than DAmB, is still associated with a significant risk of serious AEs [8]. This risk is of particular concern in low-resource settings, where the ability to monitor and respond to AEs is less robust.

To date, no systematic review has tried to summarize AE data associated with single high-dose LAmB across trials. We conducted a systematic review and meta-analysis to pool data from the existing literature to determine if treatment with a single high-dose LAmB, when used for induction treatment of invasive fungal infections in people with HIV, carries a lower risk of serious adverse events compared with treatment with a traditional WHO-approved formulation or dosing of amphotericin B.

## METHODS

### Search Strategy and Study Inclusion Criteria

We searched Medline (PubMed), Scopus, and the Cochrane Library (Wiley) from inception to April 2023 for relevant studies. We included studies that (1) compared single high-dose LAmB with alternative WHO guideline–recommended induction amphotericin B regimen for treatment of invasive fungal infection in people with HIV; (2) reported serious or life-threatening (grade 3 and 4) adverse events for each group at the conclusion of, or shortly after, induction therapy; and (3) were published in English. We assessed AEs at a minimum of 2 weeks after start of treatment to account for all AEs that may be seen during the entirety of induction therapy (2 weeks). One study [2] reported AEs at 3 weeks and not at 2 weeks; thus AEs at 3 weeks were assessed for that study. Studies published before LAmB was approved by the US Food and Drug Administration (1997) were excluded. The primary outcome was the rate of overall grade 3 and 4 AEs between single high-dose LAmB and the control group. The secondary outcome was 10-week mortality. The protocol was registered at PROSPERO (CRD42023424942) before data extraction.

To locate relevant studies in Medline, we used exploded MeSH terms and keywords to generate sets for the following themes: liposomal amphotericin B, HIV infection, administration, and dosage. We then used the Boolean term “AND” to find their intersection. The search strategy was designed with the support of a biomedical librarian (H.B.B.). This basic approach was modified to search each electronic database. Details of the structure and findings of each electronic search strategy can be found in the Supplementary Data (Supplementary Appendix 1).

Abstracts were screened based on title and abstract by 2 investigators independently (H.K., C.C.H.) using Rayyan [9]. Full-text review to select studies for inclusion was performed by 2 investigators independently (H.K., C.C.H.). Any discrepancy was resolved by a third investigator (R.A.M.).

### Data Extraction and Risk of Bias Assessment

Full-text data extraction and risk of bias assessment were performed independently by 2 investigators (H.K., J.P.U.). A standardized data collection form was used to abstract relevant information from included studies such as clinical outcomes and risk of bias. One study included in the analysis [3] was not yet published at the time of our data extraction, and the author (A.C.P.) was contacted for 2-week AEs and 10-week mortality data. The Cochrane Risk of Bias 2 tool was used to assess risk of bias [10].

### Publication Bias

As a result of the small number of studies identified, a formal funnel plot is not included. Instead, we used a qualitative approach to comment on whether publication bias was a potential risk among included studies.

### Data Synthesis and Analysis

After data extraction, we used a random-effects model to calculate pooled risk ratios (RRs) with 95% CIs using RevMan 5.4 [11]. We chose to report RRs instead of odds ratios, as risk is generally more familiar to health professionals and the public and less complicated to interpret than odds [12]. We used a “moment-based” approach, specifically the Mantel-Haenszel method, to calculate between-study variation. The Mantel-Haenszel method contrasts each study result with that of a Mantel-Haenszel fixed-effect meta-analysis and is more robust when there are few events or small samples [13]. We assessed whether there was evidence of heterogeneity across study results. We defined statistically significant and meaningful heterogeneity as a *P* value <.10 and inconsistency (*I*^2^) >50%.

To calculate the risk ratios for our primary outcome, we used the following approach. Our numerator included the number of people experiencing grade 3 and 4 AEs in the study group. Our denominator included the total number of people in that study group who were at risk for the outcome. The method of grading AEs was noted. One paper [15] did not report the overall number of grade 3 or 4 AEs experienced by participants following the induction treatment, so we added individually reported grade 3 and grade 4 AEs to calculate the overall number of AEs for this study.

We categorized individually reported AEs into 3 categories: renal (creatinine increase, hypokalemia, hypomagnesemia, and hypernatremia), hematological (anemia, thrombocytopenia, neutropenia), and infusion-related (phlebitis, infusion reaction). One study [3] did not use severity grading for infusion-related AEs; therefore, we reported infusion-related AEs of any severity across all studies. We performed a subgroup analysis looking at any grade 4 events as well as grade 4 events that were classified as renal or hematological.

Our secondary outcome included 10-week mortality. When data were available on 10-week mortality, we selected the intention-to-treat results for the analysis.

## RESULTS

**Figure 1.**
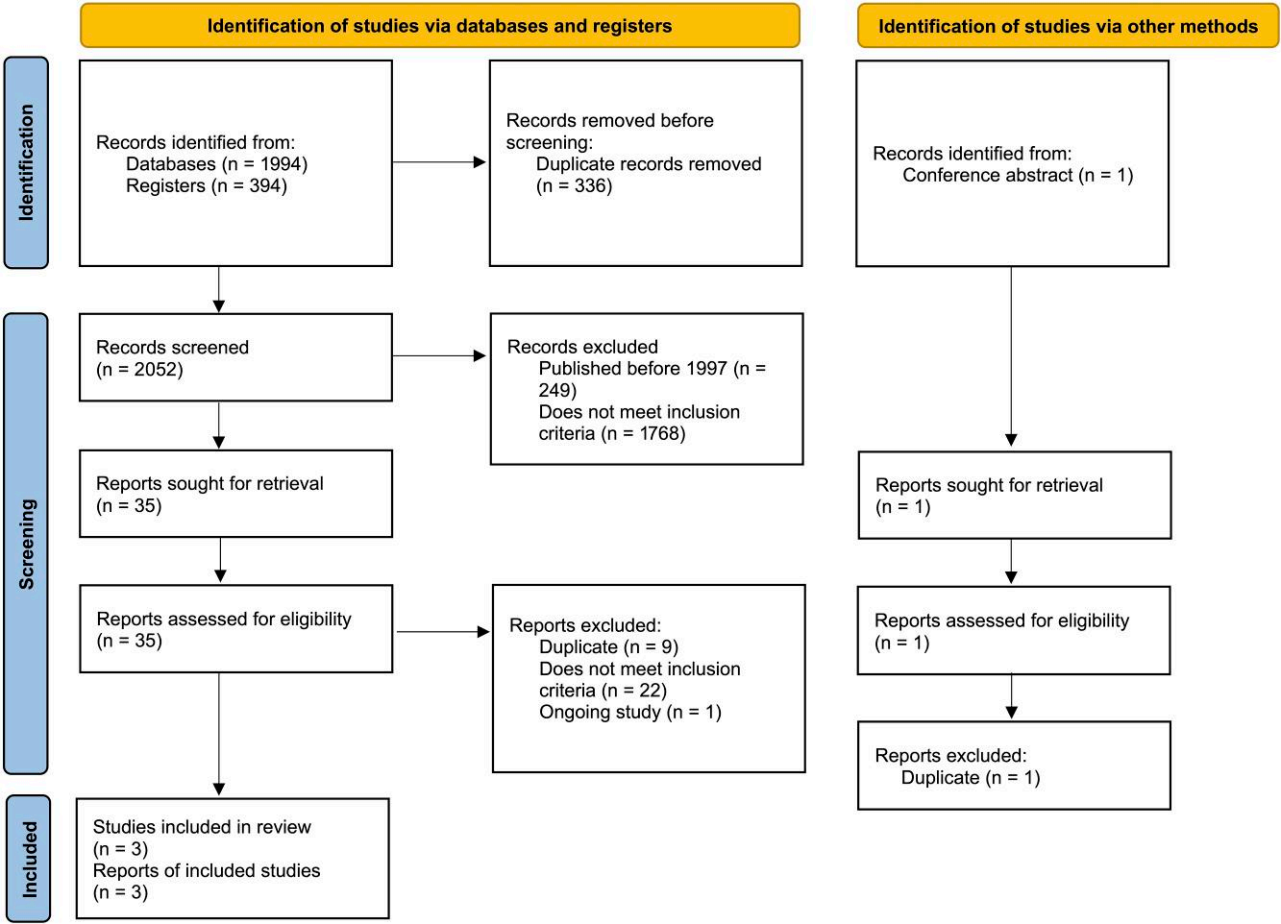
PRISMA flow diagram of included studies [14]. Abbreviation: PRISMA, Preferred Reporting Items for Systematic Reviews and Meta-Analyses.

After duplicate removal, 2052 abstracts were screened based on title and abstract. Thirty-five citations were selected for full-text review, and 3 studies were included (Figure 1). The characteristics of the included studies are summarized in Table 1. One large study [2] compared single high-dose LAmB with DAmB, and 2 smaller studies [3, 15] compared single high-dose LAmB with 14 days of LAmB. The number of concurrent antifungal agents used as induction therapy ranged from 0 to 2. One of the included studies was unpublished at the time of screening and has subsequently been published [3].

**Table 1.** Included Studies and Their Characteristics

Study	Design	Study Location	Fungal Infection	Intervention Group Size and Regimen	Control Group Size and Regimen	AE Grading and Timing of Assessment
Jarvis 2019 [15]	RCT	Botswana, Malawi, South Africa, Uganda, Zimbabwe	Cryptococcal meningitis	n = 16 LAmB 10 mg/kg on D1 Fluconazole 1200 mg QD × 14d	n = 17 LAmB 3 mg/kg/d × 14 d Fluconazole 1200 mg QD × 14 d	DAIDS, 2 wk
Jarvis 2022	RCT	Botswana, Tanzania	Cryptococcal meningitis	n = 420 LAmB 10 mg/kg on D1 Flucytosine 100 mg/kg/d × 14 d Fluconazole 1200 mg QD × 14 d	n = 422 DAmB 1 mg/kg/d on D1–D7 Flucytosine 100 mg/kg/d on D1–D7 Fluconazole 1200 mg QD on D8–D14	DAIDS, 3 wk
Pasqualotto 2023	RCT	Brazil	Histoplasmosis	n = 34 LAmB 10 mg/kg on D1	n = 37 LAmB 3 mg/kg/d × 14 d	US FDA, 2 wk

Abbreviations: AE, adverse event; DAmB, amphotericin B deoxycholate; D, day; DAIDS, Division of Acquired Immunodeficiency Syndrome; LAmB, liposomal amphotericin B; RCT, randomized controlled trial; US FDA, United States Food and Drug Administration; QD, quaque die (Latin for once a day).

Risk of bias assessment is available for review in the Supplementary Data (Supplementary Appendix 2). Overall, the concern for bias was low for individual studies, especially given the objective outcomes studied. It is unlikely that we are missing small studies with negative results given the comprehensive literature search undertaken. Therefore, the risk of publication bias was deemed to be low.

**Figure 2.**
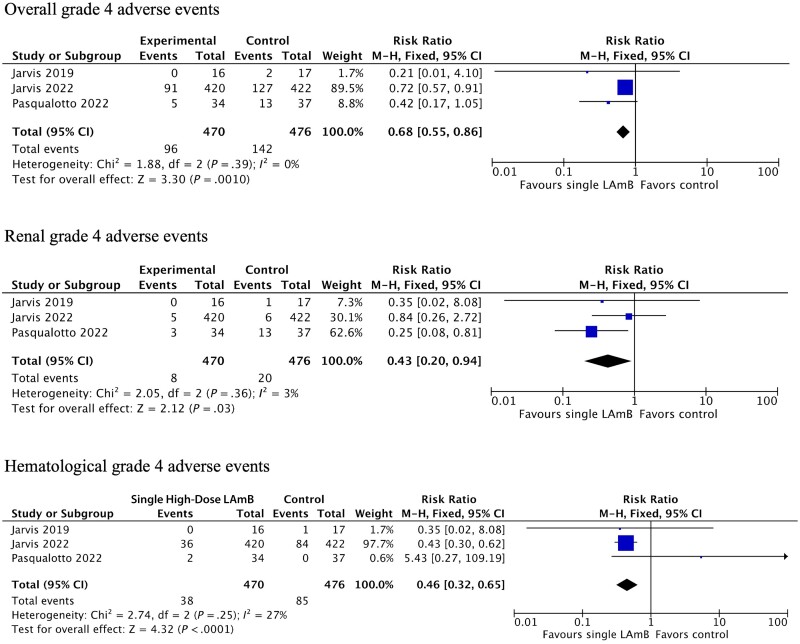
Single high-dose LAmB vs traditional amphotericin induction therapy grade 3 and 4 adverse events meta-analysis. Infusion-related adverse events are of all severities. Abbreviation: LAmB, liposomal amphotericin B.

Compared with controls, patients who received single high-dose LAmB were less likely overall to develop grade 4 AEs (RR, 0.68; 95% CI, 0.55–0.86), grade 4 renal AEs (RR, 0.43; 95% CI, 0.20–0.94), or grade 4 hematological AEs (RR, 0.46; 95% CI, 0.32–0.65), the most severe, life-threatening AEs (Figure 2).

**Figure 3.**
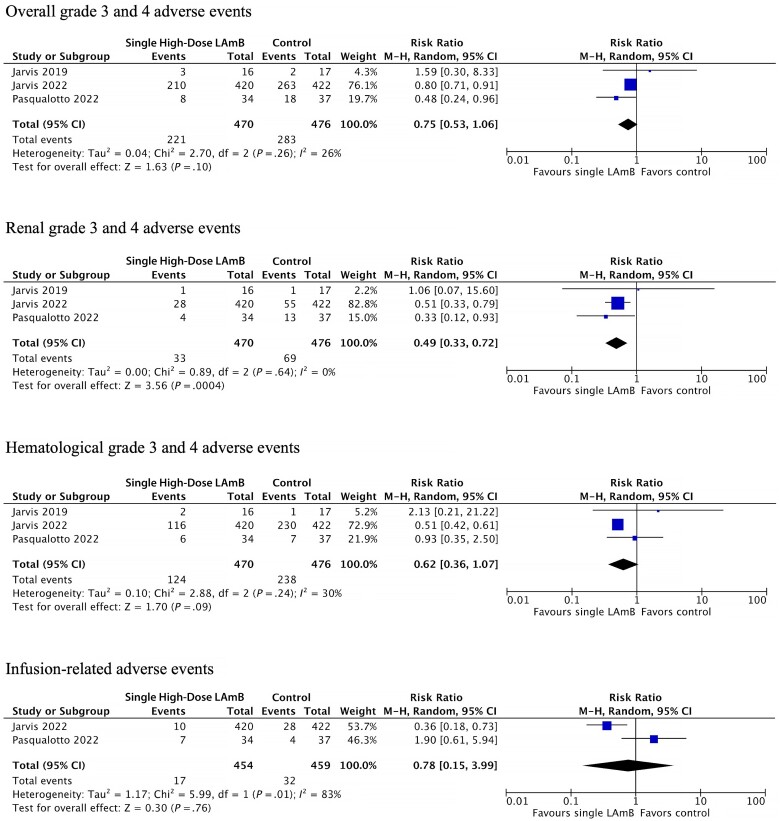
Single high-dose LAmB vs traditional amphotericin induction therapy 10-week mortality meta-analysis. Abbreviation: LAmB, liposomal amphotericin B.

Patients who received single high-dose LAmB were as likely to develop the combined outcome of grade 3 or 4 AE (RR, 0.75; 95% CI, 0.53–1.06), grade 3 and 4 hematological AE (RR, 0.62; 95% CI, 0.36–1.07), and infusion-related AE of any severity (RR, 0.78; 95% CI, 0.15–3.99). Those who received single high-dose LAmB were less likely to develop grade 3 and 4 renal AEs (RR, 0.49; 95% CI, 0.33–0.72) (Figure 3).

**Figure 4.**
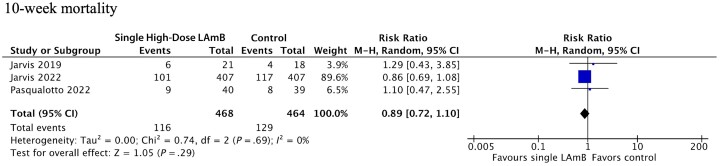
Single high-dose LAmB vs traditional amphotericin induction therapy grade 4 adverse events meta-analysis. Abbreviation: LAmB, liposomal amphotericin B.

Ten-week mortality between the 2 groups did not differ (RR, 0.89; 95% CI, 0.72–1.10) (Figure 4). Heterogeneity was low across studies, except for infusion-related AEs.

## DISCUSSION

Our systematic review and meta-analysis found that single high-dose LAmB—as induction therapy for cryptococcal meningitis or disseminated histoplasmosis in people with HIV—was associated with a lower risk of life-threatening (grade 4) adverse events compared with alternative WHO-recommended amphotericin-based regimens. This difference was driven by a reduction in grade 4 renal and hematologic adverse events.

Our findings may be particularly pertinent for routine hospital settings in low- and middle-income countries (LMICs), where the ability to monitor and respond to severe AEs is less robust. Furthermore, in such settings, there may be lower adherence to strategies to minimize amphotericin B–associated toxicities [16]. In 1 observational study from hospitals in South Africa, toxicities associated with amphotericin B deoxycholate in HIV-associated cryptococcal disease were very frequent [16]. Yet only two-thirds of patients had baseline hemoglobin, potassium, and creatinine checked, only 40% received preemptive hydration, and <20% received optimal potassium supplementation [16]. The researchers found that patients who received appropriate prehydration before amphotericin B deoxycholate were 70% more likely to survive to discharge. Avoiding serious AEs may be critical to surviving hospitalization in LMICs, making the safety advantage of single high-dose LAmB potentially greater in these settings.

Our findings may also have implications for using single high-dose LAmB in induction therapy in well-resourced contexts, where there is growing advocacy for this dosing strategy [17]. One limiting factor is the absence of a large randomized clinical trial in a high-income country comparing induction therapy involving single high-dose LAmB with existing standard of care using longer duration and a higher total dose of LAmB for HIV-associated cryptococcosis [18] or histoplasmosis [19]. Despite this, our findings add reassurance that single high-dose LAmB is a safe option and may increase uptake of this treatment strategy. In the short run, the use of single high-dose LAmB as antifungal induction therapy in well-resourced settings may have a role for certain patients; for example, it may be considered for patients with cryptococcal meningitis and HIV who are unwilling to remain in the hospital for a 2-week period.

The pharmacodynamic and pharmacokinetic differences between LAmB and DAmB may explain their differing safety profiles and provide reassurance regarding the efficacy of single high-dose LAmB. LAmB is released more slowly than DAmB, with a lower initial volume of distribution [20]. Less LAmB is excreted via urine or feces compared with DAmB [20]. As amphotericin B can induce pro-inflammatory cytokines and vasoconstrict afferent renal arterioles [20], fewer AEs are expected from LAmB's slower release and lower renal excretion.

The pharmacodynamic index most predictive of amphotericin efficacy is the ratio of maximal concentration to minimum inhibitory concentration (Cmax/MIC) [21]. The liposomal vesicles in LAmB are small and negatively charged, allowing the vesicles to avoid recognition and uptake by the mononuclear phagocyte system. This results in a single dose of LAmB yielding a much higher Cmax and a much larger area under the curve compared with DAmB [20], which is supportive of its clinical efficacy. The AMBITION trial demonstrated noninferior cerebrospinal fluid fungal clearance with single high-dose LAmB compared with DAmB [2].

The strengths of our study include a comprehensive search of the literature that included conference abstracts and other unpublished literature. Another strength is the biological plausibility: Considering that the total exposure to amphotericin B is lower with the single, high-dose LAmB strategy, it is not surprising that there is less toxicity. We also observed low heterogeneity in most adverse event categories, supporting a consistency of effect. Although the Pasqualotto study excluded patients with creatinine >1.5 times the upper limit of normal while the 2 Jarvis studies did not exclude for renal dysfunction, reassuringly only 5 out of 882 participants in the Pasqualotto study were excluded due to high creatinine. This suggests that there may be low heterogenicity in the baseline patient characteristics across trials, despite the fact that they had different fungal infections.

Our study has several limitations. First, as a result of a large number of patients included in 1 study comparing single high-dose LAmB with DAmB [2], our results are weighed in the direction of this study. However, the lower rate of grade 4 AEs was consistent across all included studies. Second, only a single pilot study studying disseminated histoplasmosis in HIV infection was identified. Additional data in this specific domain are anticipated in the years ahead from a large planned study. Third, single high-dose LAmB was used alone or in combination with 1 or 2 antifungal agents as induction therapy in the 3 included trials, which may contribute to heterogeneity.

Cost is an important driver of antifungal use in LMICs. For example, in South Africa in 2016, the cost of LAmB was US$160 per 50-mg vial, >60 times the cost of amphotericin B deoxycholate [16]. The single high-dose LAmB strategy offers an obvious financial advantage over 14 days of LAmB. The cost-effectiveness analysis from the AMBITION trial showed that there is a financial advantage of single high-dose LAmB compared with DAmB [7]. Our study findings of safety benefits of single high-dose LAmB may increase utilization of this strategy, which may help lessen the financial burden of treating invasive fungal infections in people with HIV.

## CONCLUSIONS

This study adds further evidence to support the use of single high-dose LAmB over other alternative regimens to treat cryptococcal meningitis or histoplasmosis in people with HIV in resource-limited settings. Single high-dose LAmB is safer than traditional amphotericin B–based regimens when used for induction therapy to treat cryptococcal meningitis and histoplasmosis in people with HIV.

## Supplementary Material

ofad472_Supplementary_Data

## Supplementary Data

Supplementary materials are available at *Open Forum Infectious Diseases* online. Consisting of data provided by the authors to benefit the reader, the posted materials are not copyedited and are the sole responsibility of the authors, so questions or comments should be addressed to the corresponding author.
