# Supplementary material for: Safety of Single High-Dose Liposomal Amphotericin B for Induction Treatment of Cryptococcal Meningitis and Histoplasmosis in People With HIV: A Systematic Review and Meta-analysis
Source: Open Forum Infect Dis. 2023 Sep 20;10(10):ofad472. doi: 10.1093/ofid/ofad472 (PMC10551850; doi:10.1093/ofid/ofad472)
Supplement: ofad472_Supplementary_Data [file ofad472_supplementary_data.docx]

**Supplementary material**

**Appendix 1:**

**Database:** Medline via PubMed

**Dates covered:** since inception to last date searched

**Date of search:** 4/25/23

**Search Terms/Results:**

|  | Query | Results |
| --- | --- | --- |
| #1 | “Amphotericin B” [Mesh] OR Amphotericin B[tiab] OR AmBisome[tiab] OR L-AmB[tiab] OR LAmB[tiab] | 37,452 |
| #2 | “HIV infections” [Mesh] OR HIV[tiab] OR Human immunodeficiency virus[tiab] | 442,039 |
| #3 | "administration and dosage" [Subheading] OR Dose[tiab] OR Dosage[tiab] OR Dosing[tiab] OR Course[tiab] OR Formulation*[tiab] OR mg/kg[tiab] | 3,435,904 |
| #4 | #1 AND #2 | 2,078 |
| #5 | #4 AND #3 | 728 |

**Database:** Scopus

**Dates covered**: since inception to last date searched

**Date of search**: 4/25/23

**Search Terms/Results**:

|  | Query | Results |
| --- | --- | --- |
| #1 | “Amphotericin B” OR AmBisome OR L-AmB OR LAmB | 120,663 |
| #2 | “HIV infections” OR HIV OR Human immunodeficiency virus | 448,884 |
| #3 | "administration and dosage" OR Dose OR Dosage OR Dosing OR Course OR Formulation* | 5,310,519 |
| #4 | (“Amphotericin B” OR AmBisome OR L-AmB OR LAmB) AND (“HIV infections” OR HIV OR Human immunodeficiency virus) | 4,437 |
| #5 | (“Amphotericin B” OR AmBisome OR L-AmB OR LAmB) AND (“HIV infections” OR HIV OR Human immunodeficiency virus) AND ("administration and dosage" OR Dose OR Dosage OR Dosing OR Course OR Formulation*) | 1,179 |

**Database:** The Cochrane Library (Wiley)

**Dates covered**: since inception to last date searched

**Date of search**: 4/25/23

**Search Terms/Results**:

|  | Query | Results |
| --- | --- | --- |
| #1 | “Amphotericin B” OR AmBisome OR L-AmB OR LAmB | 1,446 |
| #2 | “HIV infections” OR HIV OR Human immunodeficiency virus | 30,703 |
| #3 | "administration and dosage" OR Dose OR Dosage OR Dosing OR Course OR Formulation* | 560,649 |
| #4 | (“Amphotericin B” OR AmBisome OR L-AmB OR LAmB) AND (“HIV infections” OR HIV OR Human immunodeficiency virus) | 136 |
| #5 | (“Amphotericin B” OR AmBisome OR L-AmB OR LAmB) AND (“HIV infections” OR HIV OR Human immunodeficiency virus) AND ("administration and dosage" OR Dose OR Dosage OR Dosing OR Course OR Formulation*) | 87 |

**Appendix 2.** Risk of bias assessment

|  | Selection bias: random sequence generation | Selection bias: allocation concealment | Performance bias: blinding of subjects and assessors | Detection bias: blinding of outcome assessment | Attrition bias: incomplete data | Reporting bias: selective reporting |
| --- | --- | --- | --- | --- | --- | --- |
| Jarvis 2019 | Low | Low | Low* | Low | Low | Low |
| Jarvis 2022 | Low | Low | Low* | Low | Low | Low |
| Pasqualotto 2022 | Low | Unable to assess | Low* | Low | Low | Low |

* Performance bias was thought to be less likely given the hard outcomes studied.
